# Decision avoidance and post-decision regret: A systematic review and meta-analysis

**DOI:** 10.1371/journal.pone.0292857

**Published:** 2023-10-13

**Authors:** Qing Han, Susanne Quadflieg, Casimir J. H. Ludwig

**Affiliations:** 1 School of Psychological Science, University of Bristol, Bristol, United Kingdom; 2 Department of Social Policy and Intervention, University of Oxford, Oxford, United Kingdom; University of Klagenfurt, AUSTRIA

## Abstract

Decision Avoidance (DA) strategies allow people to forego or abandon effortful deliberation by postponing, bypassing, or delegating a decision. DA is thought to reduce regret, primarily by allowing decision makers to evade personal responsibility for potential negative outcomes. We tested this relation between DA and post-decision regret in a multilevel meta-analysis of 59 effect estimates coming from 13 papers. Five DA strategies were considered: status quo preservation, action omission, inaction inertia, choice delegation and choice deferral. Across all effects and DA strategies, there was a non-significant trend toward DA reducing regret (Hedges’ *g* = -0.23, *p* = 0.063). When assessing individual strategies, we found that only status quo preservation reduced regret reliably (Hedges’ *g* = -0.45, *p* = 0.006). The relationship between DA and regret was unclear for the other DA strategies. We tested a number of moderators for the effect. Only ‘previous experience’ (i.e., the outcome of a previous decision) influenced the relation between DA and regret reliably. That is, if participants choose the DA option when the same choice previously led to a negative outcome, regret is actually enhanced. Overall, there is clear evidence that status quo preservation can reduce regret, but it is currently unclear whether the same holds for other DA strategies.

## Introduction

When people are shopping for orange juice in the supermarket, they may pick up the one they bought last time and do not even notice a new brand of juice right next to it. Because the previous choice is good enough, it is not necessary to spend more time choosing a juice. If so, people have engaged in what has previously been termed a decision avoidance (DA) strategy [1, 2].

Over the last decades, behavioural scientists have identified and described various DA strategies. These strategies generally result in the foregoing or abandoning of effortful deliberation by postponing, bypassing, or delegating a decision [1, 2]. Contemporary research on DA strategies can be traced back to the earlier concept of decision attitude. This concept was originally used to highlight that people can differ in their “desire to make or avoid decisions” [3], but it failed to promote systematic research on DA strategies. To overcome this empirical lacuna, a seminal model by Anderson [1] proposed to identify and compare such strategies in terms of their cognitive and emotional commonalities. Based on this suggestion, efforts to identify common DA strategies remain ongoing. Otto and colleagues [2], for example, recently coined the term decision side-stepping to draw attention to DA strategies that rely on a choice precedent (e.g., in the form of existing norms and/or previous decisions).

Though a comprehensive taxonomy of DA strategies remains to be established, there is little doubt that well-known DA strategies shape a wide range of human behaviours including purchasing decisions, election outcomes and public policy commitments [4, 5]. Indeed, many contemporary “nudge” interventions capitalise on DA strategies by making socially desirable decisions a default option so that committing to this option becomes less effortful than circumventing it [6]. For example, several European countries use the “opt-out system” for organ donation, which means residents are presumed as a donor by default unless they submit an active opt-out request. In these countries, about 90% of people are listed as organ donors. In contrast, in “opt-in” European countries, this number is only around 15% [7]. Additionally, a recent review on the topic found that the consent rate in “opt-out” organ donation countries increased by 21% to 76% over a period of 5 to 14 years [8].

Although DA strategies are widely used and exploited in daily life, much remains to be learnt about their psychological "benefits" beyond avoiding the cognitive cost of effortful decision making. Identifying these additional psychological benefits is of particular scientific relevance as it promises to advance our understanding of the strategies’ frequent use [9]. In this context, it has been argued that people may choose to avoid effortful decisions in order to minimise feelings of regret that could arise from making a sub-optimal decision [1]. Based on this suggestion, the current meta-analysis explored the relation between using a DA strategy when making a decision and feeling post-decision regret.

Several studies provide evidence that decisions involving DA strategies induce less post-decision regret than decisions that involve more active deliberation [10–13]. Nevertheless, some studies have also reported regret intensification following DA (e.g., studies 2 and 3 in Abendroth & Diehl, 2006 [14]; studies 1, 2 and 4 in Inman & Zeelenberg, 2002 [15]; or study 2 in Sevdalis et al., 2006 [16]). Comparing these contradictory results remains difficult as most studies have examined just one specific, but not necessarily the same, DA strategy. As a consequence, it is uncertain whether a general link between DA and regret evasion exists or whether such a link may be strategy-dependent. Moreover, studies vary along a number of other dimensions which may influence the strength of the relation between DA and regret. Therefore, we conducted a meta-analysis that integrated empirical findings from 13 published papers [11–23], with the aim to: (1) examine the existence and strength of a systematic link between DA and post-decision regret; (2) examine the heterogeneity of the link between DA and regret across different DA strategies; and (3) if we find a link between DA and regret, we would like to further explore pivotal moderator variables of the link between DA and regret.

### Common decision avoidance strategies

We included five well-researched DA strategies in our analysis, namely status quo preservation [24, 25], action omission [26], choice deferral [27], choice delegation [28], and inaction inertia [25, 29]. *Status quo preservation* describes people’s preference to choose options that are likely to maintain the current state of affairs [24]. A well-known example of this strategy comes from the soft drink industry. When Coca Cola developed their “New Coke” formula in 1985, it was preferred by consumers in blind tests, but once released on the market most people stuck to the original Coke and New Coke was soon discontinued [30]. Thus, even though customers were offered an appealing new drink, many of them simply adhered to their established purchasing behaviour.

A closely related, but ultimately distinct DA strategy is *action omission* [26]. This strategy entails choosing an option that requires no action, irrespective of whether doing so maintains or changes the status quo [31]. For example, when renting a new apartment, some tenants may skip the option of choosing the best energy supplier for their own needs and simply stick with their landlord’s original choice of supplier. In this case, the tenants’ omission to act typically ends up preserving the status quo. But doing nothing can also change the status quo. Because many energy suppliers adopt a fixed-term tariff model, they can easily switch consumers to different, less attractive plans at the end of their initial service period. In this case, inactive tenants normally end up facing a change in status quo.

*Choice deferral* refers to a DA strategy through which people temporarily decide not to choose any of the available alternatives [1, 32, 33]. This strategy is often used when people are confronted with several attractive options and struggle to establish a clear preference for any one [34, 35]. For example, a consumer may be happy to buy any one of two attractive products when they are presented in isolation. However, when both products are presented together, the same consumer may feel unable to decide between them and postpone the decision, often in the hope that a clearer preference will form down the line [36]. *Choice delegation* captures a DA strategy through which a decision-maker assigns the responsibility of having to choose among viable alternatives to another agent [28, 37]. For example, many people delegate their financial choices to a financial adviser. However, more mundane examples of choice delegation, without an imbalance of expertise, come to mind easily (e.g., letting your friend decide which film to get tickets for).

*Inaction inertia*, finally, refers to the observation that after bypassing an initial attractive action opportunity, people are less likely to choose a similar, but less attractive option, even when that option is still better than some prior “baseline” [25, 38, 39]. For instance, customers are more likely to reject a table with a 10% discount if they have already missed the opportunity to buy the same table with a 15% discount [40, 41].

Importantly, all of the DA strategies listed above have in common that they produce some form of decision outcome, even if it is simply the choice to postpone a decision. In this regard, they do not help to avoid decision-making altogether, but they specifically help to circumvent effortful decision-making. Of course, the different strategies may not all be equally effective at avoiding active deliberation or effortful action. For instance, choice deferral can sometimes arise after a period of deliberation, at which point the decision maker concludes that they are not yet ready to commit to a specific choice. Nevertheless, all strategies count as DA strategies because they save some degree of cognitive effort or time during the decision-making process. At this point, however, it remains uncertain whether and to what extent they can also bestow a shared psychological benefit in terms of regret evasion. It is possible, after all, that regret-related effects could be limited to strategy-dependent decision outcomes and/or mechanisms [1, 2]. To address this possibility, the current meta-analysis considered the regret-related effects of several different DA strategies jointly as well as separately.

### Decision avoidance and regret

Feelings of regret have been identified as one of the primary negative emotions that people seek to avoid when making decisions [42–44] (for a review see Connolly & Zeelenberg, 2002 [45]). With this finding in mind it has been proposed that many, if not most DA strategies facilitate regret evasion by allowing decision-makers to re-direct blame for disadvantageous outcomes to sources other than themselves [2]. Common alternative sources for blame include existing norms (e.g., in the case of status quo preservation), prior decisions (e.g., in the cases of action omission or inaction inertia), or other agents (e.g., in the case of choice delegation). In line with the suggestion’s intuitive appeal, empirical evidence in support of a systematic link between DA and regret evasion has accumulated in recent years. But there is also growing evidence that the proposed link may be stronger for some DA strategies than others.

In terms of status quo preservation, for example, it has long been argued that people experience less regret when they make a decision that maintains rather than changes the status quo [46]. This claim has received extensive empirical support [11, 12, 15, 18] and evidence to the opposite remains scarce (but see Bar-Eli et al., 2007 [47]). In contrast, with regard to action omission, empirical evidence is less consistent. Some studies have reported a reduction in regret following action omission [19, 48–52], but others have found the opposite pattern of results (e.g., Connolly & Reb, 2003 [53]; study 2 of Sevdalis et al., 2006 [16]) or suggest that action omission can have contradictory effects on short-term and long-term regret [14, 54, 55].

The latter observation is particularly noteworthy as it suggests that the link between DA and regret evasion may not only be strategy-dependent, but could also vary due to additional moderator variables such as at what point during the decision-making process regret is being measured. Therefore, we decided to catalogue such variables in our survey of the literature. First, as already noted, the timing of regret measurement appears to be important. The regret-reducing effect of some DA strategies (such as status quo preservation) may be more pronounced before people learn about the consequences of their decision(s) rather than after [17]. Second, the regret-reducing effects of DA strategies can depend on the type of outcome they achieve. Specifically, feelings of regret are likely to increase if a DA strategy led to a negative outcome, rather than to ambiguous, neutral or positive decision outcomes [14, 19]. Third, there is evidence that the decision making history, especially experience with previous decision outcomes, can modulate the link between DA and regret evasion. For instance, using hypothetical scenarios, Zeelenberg and colleagues [15, 18] showed that preserving the status quo can cause greater feelings of regret when, in the scenario, the same option/decision previously led to a negative outcome. Finally, studies have shown that the link between DA and regret evasion may depend on age [56], gender and region [57, 58]. Taken together, a growing body of work indicates that the postulated link between DA and regret evasion may not only vary across different kinds of DA strategies, but also depend on a number of moderator variables.

### The present meta-analysis

**Fig 1:**
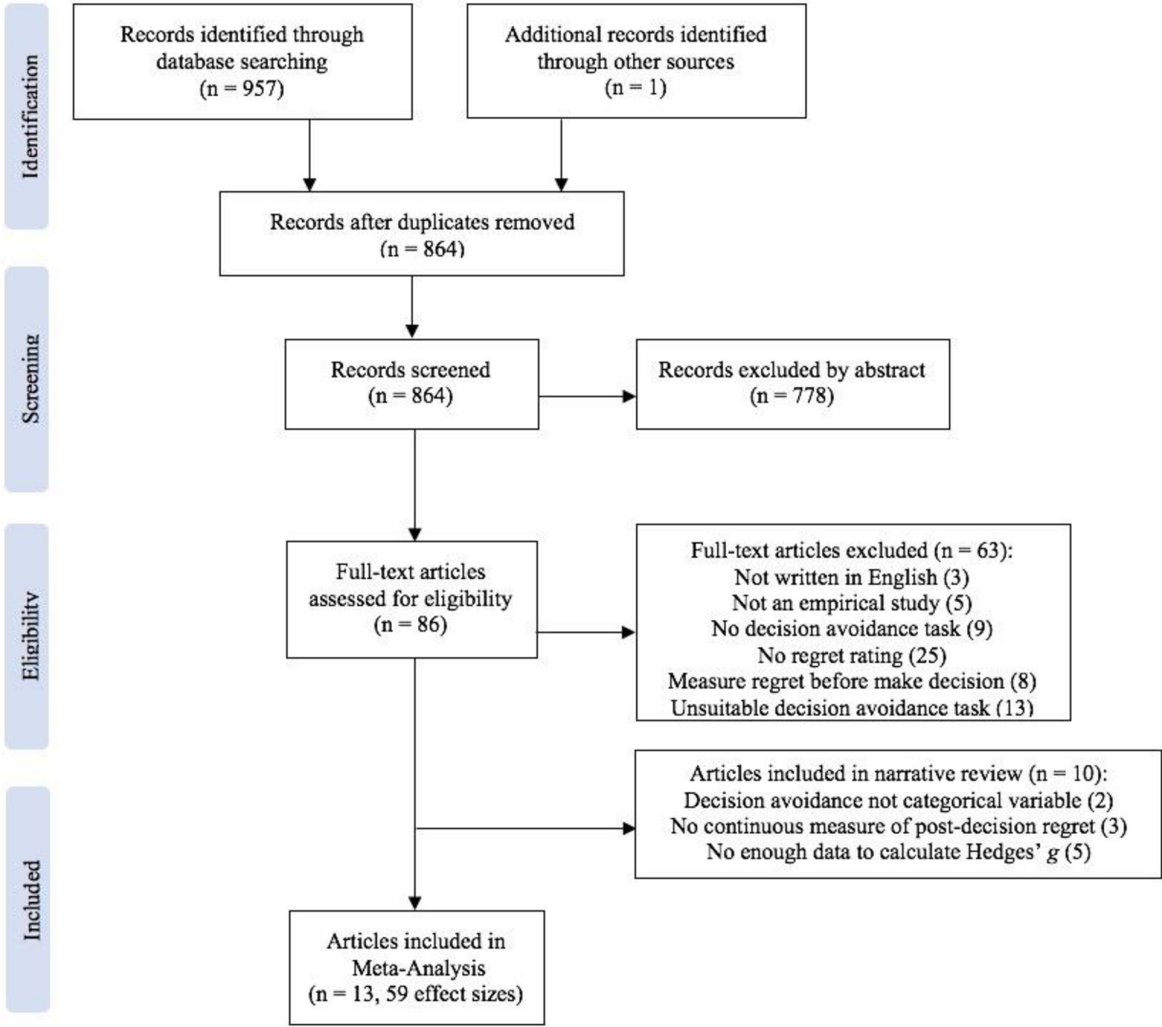
PRISMA flowchart illustrating the stages involved in screening studies for the meta-analysis.

**Fig 2:**
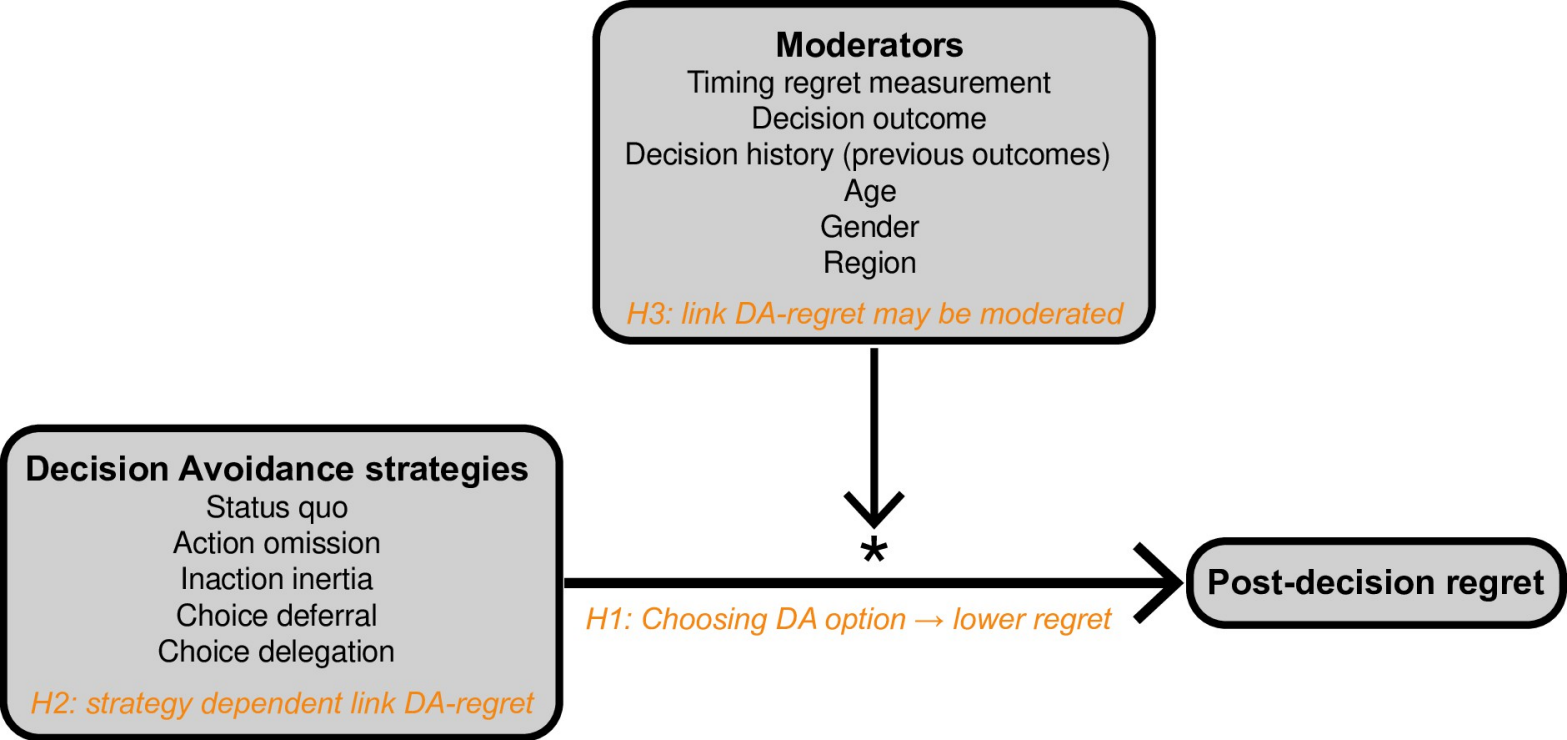
Summary of hypotheses.

Based on previous findings in the literature, we predicted that people who choose a DA strategy over a non-DA strategy during the decision-making process experience lower levels of post-decision regret (Hypothesis 1). Beyond testing this basic link, we also examined the link’s strength across different DA strategies. Specifically, we hypothesised that the link’s magnitude and/or direction might differ across DA strategies (Hypothesis 2). In addition, we explored whether a number of possible moderator variables would affect the magnitude and/or direction of the postulated link (Hypothesis 3). By testing these hypotheses, our overall goal was to advance the field’s current understanding of the relationship between DA and post-decision regret. Fig 1 shows the PRISMA flowchart for our systematic review and meta-analysis. Fig 2 shows a summary of our hypotheses, highlighting the three-tiered approach to our analyses.

## Method

### Literature search and selection

Six databases were searched for studies exploring the relationship between DA and regret: PsycInfo, PsyArticles, Web of Science (including seven databases: Web of Science Core Collection, BIOSIS Citation Index, Derwent Innovation Index, KCI-Korean Journal Database, MEDLINE, Russian Science Citation Index, SciELO Citation Index), ProQuest, SCOPUS and JSTOR. This study followed the Preferred Reporting Items for Systematic Reviews and Meta-analyses (PRISMA) reporting guidelines [59]. Two groups of keywords were used: keywords for decision avoidance (“decision avoidance” OR “status quo” OR “default bias” OR “option fixation” OR “omission” OR “inaction inertia” OR “deferr*” OR “delegat*”) and keywords for regret (“counterfactual thinking” OR “regret”). These two groups of keywords were combined using “AND”. Detailed search strategies are presented in S1 Text in S1 File. All search was limited to studies written in English and published before January 2022. In total, 957 articles were found, and one additional article was identified from the references in one of these articles. After removing duplicates, 864 articles remained. For these 864 articles the title, keywords and abstract were screened and 778 articles were removed as they were out of scope (e.g., articles that were not about decision making as such, but just contained some of the key words somewhere in the text). A total of 86 articles were extracted for full-text screening.

Articles needed to meet the following criteria for inclusion in the meta-analysis. The study (or studies) should (a) be written in English; (b) include at least one empirical study (i.e., no review or ‘pure’ theoretical articles); (c) differentiate between DA and non-DA options or conditions, where the DA option or condition corresponds to one of the five strategies described above; (d) measure regret as a continuous variable; (e) measure regret *after* a decision (for a DA or non-DA option) has been made; (f) provide enough data for computation of Hedges’ *g* [60] (i.e., means, standard deviations, and sample size for each condition). Authors who did not present enough data in their article to allow for the computation of Hedges’ *g* were contacted via email; three authors responded and provided the required data.

We found that in some papers, due to differences in experimental design, DA was not a categorical variable or regret was not measured as a continuous variable, or the data provided by the papers were insufficient to calculate Hedges’ *g*. Even though these papers focused on the relationship between DA and regret, they could not be included in the quantitative meta-analyses. There were ten such articles, and we included them in a narrative review, reported in S1 Table in S1 File. In all, of the 86 articles that were screened, 13 were included in the meta-analysis. Most articles reported several experiments or, at least, several DA–non-DA comparisons, for a total of 59 effect sizes. We extracted the mean and standard deviation of regret scores in the DA and non-DA conditions, as well as the sample size. Where data were only reported in graphical form, we used Cochrane-recommended software PlotDigitizer (www.plotdigitizer.sourceforge.net/). Detailed information of the 13 selected articles (e.g., author, year, type of decision avoidance, levels of moderators) is given in S2 Table in S1 File.

### Data analyses

#### Main effect of DA on regret

We used Hedges’ *g* to quantify the effect size as the standardised difference between two sample means, corrected for small samples [61] (see S2 Text in S1 File). The full dataset has 13 articles with 59 effect sizes and some of these effect sizes come from the same article. As such, the dataset has a multilevel structure: the higher level here is the article level (article ID), the lower level is effect ID (a unique ID given to each effect size). Therefore, we used multilevel meta-analysis for assessing the link between DA and regret (Hypothesis 1), and for the subsequent analysis of this link for specific DA strategies (Hypothesis 2). We used the R (R Core Team, 2017) package *metafor* [62] to perform this analysis [63]. All of the above data and analysis codes are available on the Open Science Framework (https://osf.io/k4e2n/).

To estimate the overall effect size, article ID and effect ID were two random effects and the model was fitted using restricted maximum likelihood. In total the resulting model has three parameters: two variance components associated with article and effect ID, and the overall effect size estimate. It is the latter that is of critical interest. In addition, the same multilevel meta-analysis was conducted separately for subgroups defined by each DA strategy (though see below; there were too few effects for some strategies).

In the full sample we have a mix of within and between-subject comparisons (23 and 36 effect sizes, respectively). Morris and DeShon [64] indicated when combining two types of effect sizes (within and between-subject) in a meta-analysis, all effect sizes should be put onto a common scale. The focus of this meta-analysis is on the difference between DA and non-DA conditions, rather than the change within a person [64]. Therefore, we treated all these effect sizes as if they came from a between-subjects design (see Equation 1 in S2 Text in S1 File). As a result, the pooled standard deviation term in the denominator of the effect size measure [60, 65] includes between-subject variability that is irrelevant in a within-subjects design [61]. We did not have sufficient information to remove this variability and compute within-subjects effect size measures (individual-level data not available at all, t-statistics only available for 6/23 within-subject effects).There is then a danger that we are underestimating the true effect size for these within-subject comparisons.

We addressed this issue in two ways. First, we conducted a sensitivity analysis in which we restricted our overall meta-analysis only to the between-subject designs. Second, we implemented a (crude) correction of the pooled standard deviation and computed the effect sizes for within-subject comparisons with and without the correction (see formulas in S2 Text in S1 File). As it turned out, this correction barely affected the effect size estimates compared to our between-subject approximations (see S1 Fig in S1 File), so we only deal with the uncorrected effect size estimates below. Note that treating within-subject effects as if they were between-subject effects is a conservative analytic choice against Type I errors.

#### Moderating effects

Six moderators were included in this study based on previous literature and available information from included articles: timing of regret measurement, outcome, previous experience, age, gender (coded as percentage of female participants) and region (as a proxy for cultural differences). We used multilevel meta-regression to examine whether and which moderators contributed significantly to variation between observed effect sizes (Hypothesis 3). Only one moderator was considered in each regression model. Significance of that moderator is tested with a Q-test against a null hypothesis that all coefficients of the moderators (or, for a categorical predictor, its levels) are zero [66, 67]. As in the above model, article ID and effect ID were considered as random effects in multilevel meta-regression. Categorical predictors were tested with treatment contrasts, with the baseline level (intercept of the regression model) set either arbitrarily (e.g., for the ‘region’ factor, we used the data from North America as our baseline) or in accordance with what seemed the most “natural” baseline (e.g., ‘no outcome’ for the ‘outcome’ factor).

#### Publication bias analysis

Given the reliance on *published* articles in this meta-analysis, it is important to assess to what extent the effects we included may be subject to publication bias. Publication bias means that the set of effects are a biased sample from a population of effects, where smaller (null) effects are less likely to be published [68]. To test whether the publication bias exists in our dataset, we assessed a funnel plot of the observed effect sizes (x-axis) and their standard errors (y-axis). In the absence of publication bias, the effect sizes should be distributed symmetrically around a “true” value. Moreover, effect size estimates from studies with a smaller sample size should scatter more widely at the bottom (more variable estimates in effect size). This spread should decrease for larger, more powerful studies which should cluster closer to the true value [69]. As such, the data points should form, approximately, a symmetrical, upward pointing triangle [70]. Visual examination of this funnel plot and Egger’s test [71] were used to test for asymmetry.

## Results

A total of 13 articles [11–23] with 59 effect sizes were included in this meta-analysis. In the dataset, no study of choice deferral met the inclusion criteria, so four decision avoidance strategies were included in this meta-analysis: status quo, omission, inaction inertia and choice delegation.

### Main effect

**Fig 3:**
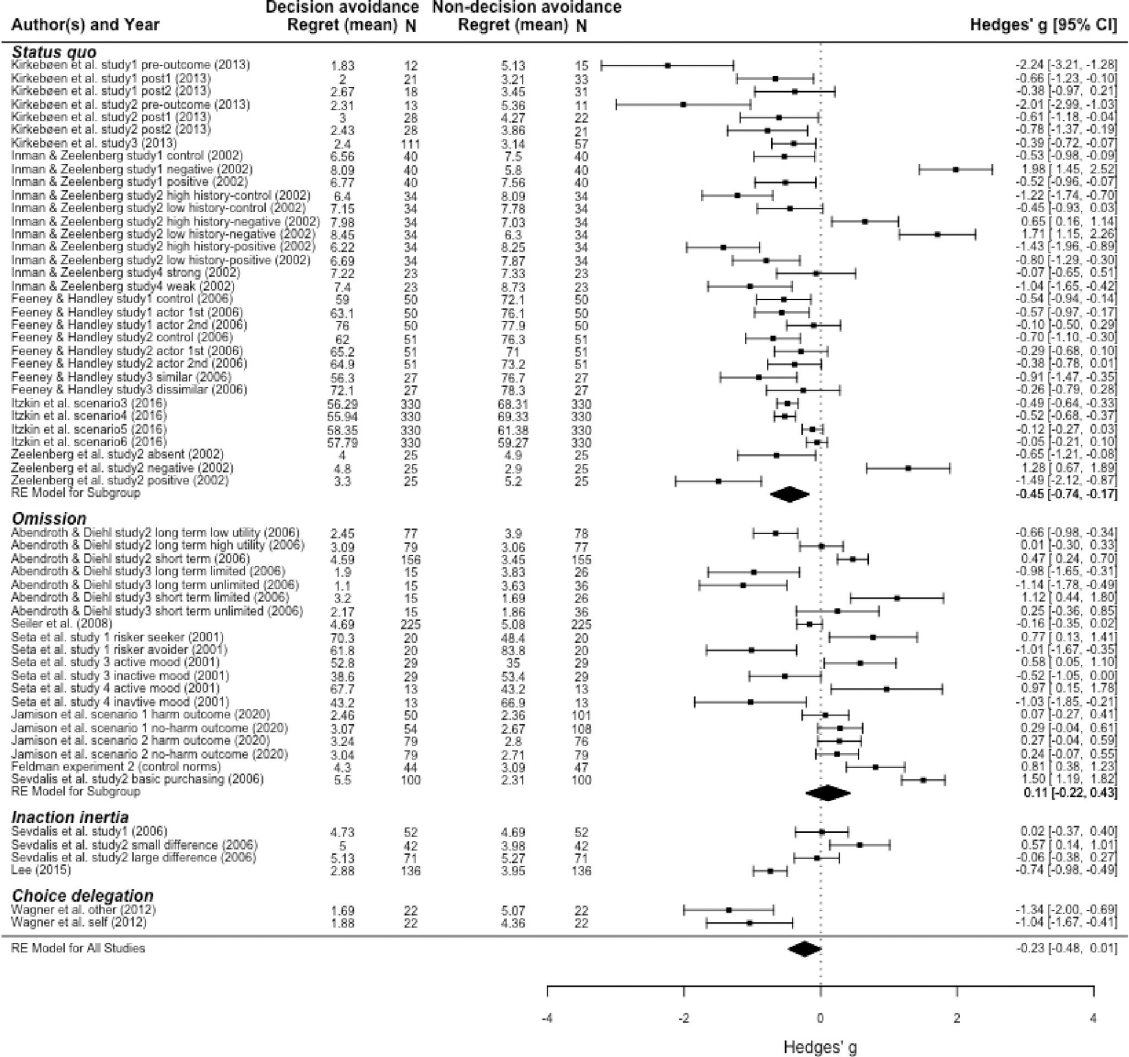
Forest plot for full sample and subgroups of status quo and omission. *Note*. Each point represents a single effect size, and the error bars represent the 95% confidence intervals.

Fig 3 shows a forest plot of the 59 effect sizes (organised by DA strategy). The diamond at the bottom of the figure indicates the overall effect size (the width of the diamond shows the 95% confidence interval). In the multilevel meta-analysis on the full dataset we found marginal evidence that participants in the DA condition had a lower level of regret than participants in the non-DA condition (k = 59, Hedges’ *g* = -0.23, 95% CI = [-0.48, 0.01], *p* = 0.063; sensitivity analysis on between-subject effects only: k = 36, Hedges’ *g* = -0.19, 95% CI = [-0.55, 0.17], *p* = 0.293). The question is now whether the direction and strength of this effect varies with DA strategy.

### Strategy-specific effects

As shown in Fig 3, too few effect sizes were available for inaction inertia and choice delegation to estimate the parameters of the multilevel meta-analytic model. Moreover, the three effect sizes of inaction inertia were extracted from two papers, and the two effect sizes for delegation were extracted from one paper. As such, a pooled effect size estimate for each of these two strategies would mainly tell us something about the strength of an effect generated by studies from a single lab. Therefore, we did not analyse these two strategies in isolation (note the missing diamonds below each of these sections in Fig 3).

The meta-analysis of the 33 effect sizes for status quo showed that regret was lower in the DA condition (maintaining status quo) compared to the non-DA condition (k = 33, Hedges’ *g* = -0.45, 95% CI = [-0.74, -0.17], *p* = 0.006). The meta-analysis of the 13 effect sizes for omission did not show a significant effect (k = 20, Hedges’ *g* = 0.11, 95% CI = [-0.22, 0.43], *p* = 0.523). Although we could not estimate an overall effect size for the remaining two strategies, it is notable that all three effects for inaction inertia appear to show an effect in the *opposite* direction to that observed for status quo and the full sample. This pattern of findings suggests strongly that the overall, marginal effect across the entire set of studies is mainly carried by the status quo strategy reducing regret. Therefore, merely being able to outsource blame for a disappointing outcome (common to all DA strategies considered here), does not seem sufficient for reducing regret.

Even just a visual inspection of the remaining three strategies grouped together suggests that the relation between DA and regret is much more mixed for this set of 26 studies. Indeed, pooling this group of studies in the multilevel model provided no evidence for a relationship between DA and regret (k = 26, Hedges’ *g* = 0.02, 95% CI = [-0.33, 0.29], *p* = 0.900). More generally, it is clear that there is substantial heterogeneity in the effect both between and within DA strategies. One question is where this heterogeneity comes from and to what extent we can account for this variability in the effect size with our chosen moderator variables.

### Moderating effects

**Fig 4:**
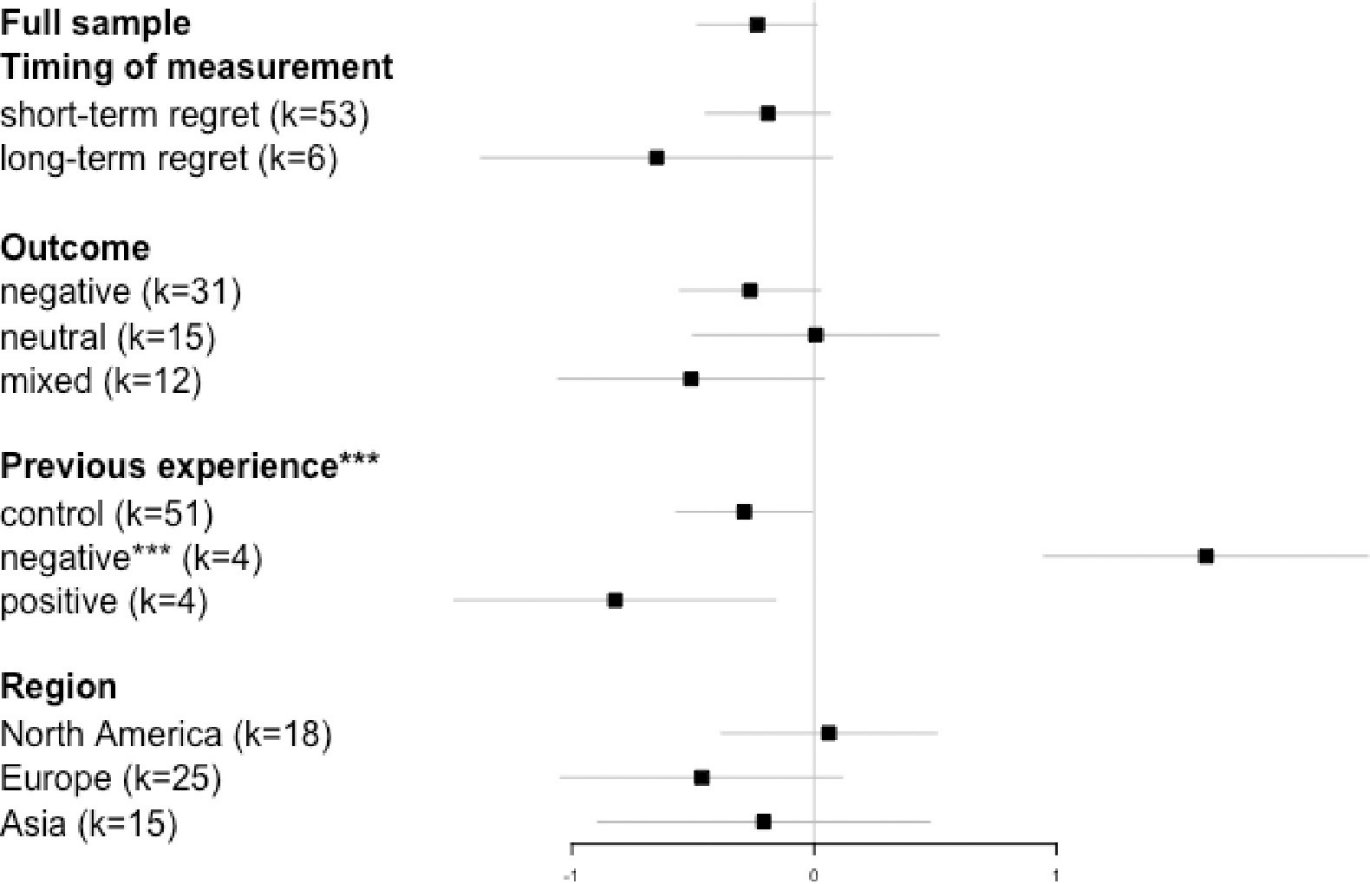
Forest plot with a meta-analytic mean and meta-regression model of moderators. *Note*. Black squares represent the estimated effect size for each level of moderators, the error bars represent the 95% confidence intervals. The first level of each moderator is set as the baseline. A significance flag * by the moderator name refers to the whole model (Q-statistic); a significance flag by each level of a predictor refers to the difference between that level and the baseline. *** *p* < 0.001. The total sample size of outcome and region is 58. One study whose outcome setting was positive was not included in the moderating analysis of outcome. For one study we could not identify the region where the study was conducted.

Fig 4 shows the moderating effect of the four categorical moderator variables (shown in bold). Below each moderator variable, we list its levels. The first level of each variable is treated as the baseline (intercept) in the regression model. Significant moderators, as determined with the Q-statistic, are flagged. For the individual levels of those moderators, a significance flag indicates a difference between that level and the baseline. The data points in Fig 4 show the pooled effect size estimate for each level of the moderators estimated from the regression model (treatment contrasts). Note that these sub-group effects may be different from 0 (as suggested by the confidence intervals), without necessarily being different from each other (as indicated by the flags). Only the latter speaks to the issue of heterogeneity.

Of the four categorical variables only *previous experience* moderated the association between decision avoidance and regret (*Q* = 43.4, *p* < 0.001). In the control condition (i.e., no previous experience or a previous neutral outcome), the association was negative (k = 51, Hedges’ *g* = -0.28, 95% CI = [-0.56, 0.00]). The treatment contrast was significant for negative prior experience but note that in this case the effect was reversed (k = 4, Hedges’ *g* = 1.40, 95% CI = [0.81, 1.98]). That is, if the previous experience was negative, DA induced *more* regret. For positive experience, the effect was negative again, but not different from the control condition (k = 4, Hedges’ *g* = -1.04, 95% CI = [-1.54, -0.54]). One concern here is that the contrast between negative previous experience and the control condition is partially confounded with DA strategy, because all the studies that manipulated previous experience (either negative or positive) involved status quo maintenance as the DA strategy. However, these effects remained significant when restricting the analysis to studies of status quo maintenance only (*Q* = 84.40, *p* < 0.001) and, indeed, when a binary DA strategy variable (status quo vs non-status quo) was included as an additional predictor (*Q* = 47.40, *p* < 0.001).

No moderating effect was found for *outcome type* (*Q* = 2.65, *p* = 0.265), *timing of regret measurement* (*Q* = 1.56, *p* = 0.212) and *region* (*Q* = 3.16, *p* = 0.207). We also tested two continuous moderators (not shown in Fig 4). Neither participant *gender* (percentage of female participants) nor average participant *age* moderated the relation between DA and regret (*β* = -0.02, *p* = 0.981; *β* = 0.03, *p* = 0.368, respectively).

### Publication bias

**Fig 5:**
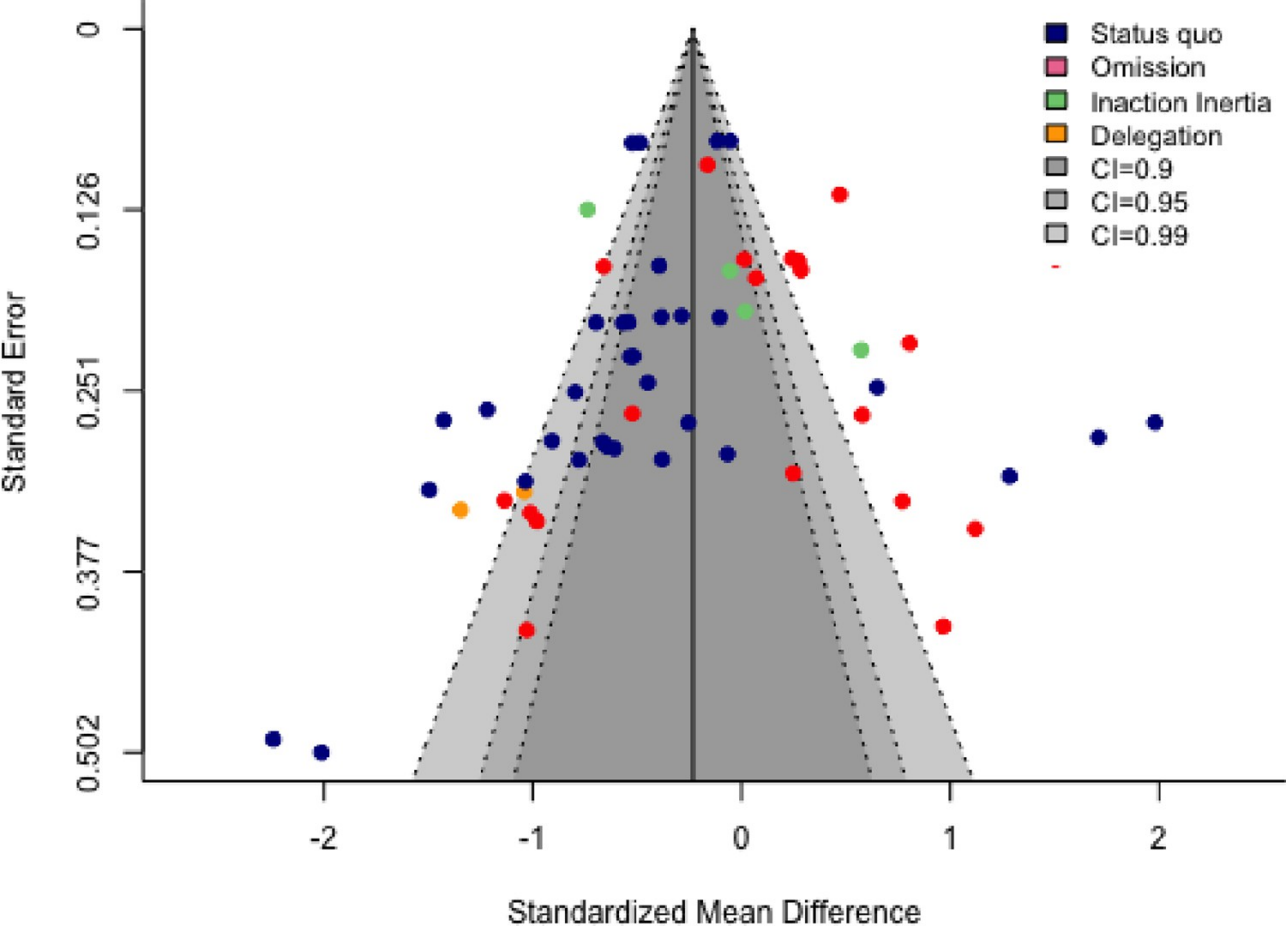
Funnel plot for full dataset. *Note*. Each point refers to an individual effect size; outer dashed lines indicate the triangular region within which 90% /95% /99% of effect sizes are expected to lie in.

Fig 5 shows the funnel plot created for the full sample, with different DA strategies marked in different colours. Egger’s test for the full sample indicates a publication bias (*z* = -2.07, *p* = 0.038). Indeed, 35/59 effects fall to the left of the overall effect of Hedges’ *g* (-0.23). Such a slight bias in favour of a more extreme (negative) association suggests that the true effect may be smaller than the overall estimate. Recall that the overall negative association between DA and regret seems to be carried mainly by the status quo strategy (Hedges’ *g* = -0.45), which is also the strategy that has been most intensively studied. Therefore, it is of interest to assess whether this effect too is potentially over-estimated in this meta-analysis. An Egger’s test of this subset of effects shows little evidence for publication bias (*z* = -1.7, *p* = 0.095).

There is another aspect to publication bias that is not assessed by the asymmetry in the funnel plot or by Egger’s test. The dark grey, grey and light grey areas of the triangle in Fig 5 show the 90%, 95%, 99% confidence intervals, respectively, for normally distributed effects around the estimated overall effect size. For sufficiently large samples and in the absence of publication bias, we would expect 90%, 95%, 99% of observed effect sizes to lie within these triangles [70]. However, Fig 5 shows quite a few effect sizes outside these triangles. It is possible that these datapoints correspond to under-powered studies that, by chance, yielded surprisingly large effects, either in the expected (negative) direction or in the unexpected (positive) direction. Such large effects might be more likely to be submitted and accepted for publication.

### Beyond quantitative assessment

Besides the quantitative meta-analysis of studies that met our strict inclusion criteria, we also conducted a qualitative synthesis of ten additional studies [10, 38, 44, 54, 72–77]. These additional studies all reported a link between DA and regret, but failed to meet one or several inclusion criteria for our meta-analysis. Specifically, they did not manipulate DA as a categorical variable, failed to measure regret on a continuous scale, and/or provided insufficient data to calculate Hedges’ g. Therefore, we simply classified each of these studies as providing (partial) support for a positive or a negative relation between DA and regret. S1 Table in S1 File provides a narrative review of this additional set of studies. Table 1 summarises the outcome of this narrative review.

**Table 1:** Summary for articles not eligible for meta-analysis.

	DA reduces regret	DA reduces regret (partial support)	DA increases regret	Total
Status Quo	5	1	0	6
Omission	0	0	3	3
Inaction Inertia	0	0	0	0
Delegation	1	0	0	1
Total	6	1	3	10

First, it is worth noting that in this set of studies status quo preservation is again the dominant strategy [10, 38, 44, 72–74]. Furthermore, in line with our findings from the quantitative meta-analysis all six studies covering this strategy provide (some) support for the observation that status quo preservation reduces regret. In the meta-analysis, the evidence for action omission reducing regret was rather mixed and considering a further three papers adds to this picture: these studies reported that action omission can actually increase rather than decrease the level of regret [54, 75, 76]. For choice delegation, only two effects (from one paper) were included in the meta-analysis and both pointed to delegation reducing regret. The one additional study in this narrative review points in the same direction [77]. To sum up, the results from our qualitative synthesis further supported the (mixed) findings from the meta-analysis. In doing so, this qualitative analysis indicates that the quantitative findings we report above are not limited to studies that fit the narrow constraints of a meta-analysis.

## Discussion

In this meta-analysis we assessed the relationship between the use of DA strategies and post-decision regret. After screening an initial set of 864 published articles, we selected 13 articles that collectively yielded 59 effect sizes (comparisons of continuous regret ratings in DA and non-DA conditions). The pooled result of all included studies indicated that people who chose the DA option had a marginally lower level of post-decision regret than those in non-DA conditions. The overall estimated effect size (Hedges’ *g*) was -0.23, which reflects a weak effect. Importantly, this overall result was largely driven by a single DA strategy known as status quo preservation, which accounted for 33/59 effects. Indeed, the subgroup analysis restricted to status quo preservation indicated a stronger result than what was found across the full set of strategies (Hedges’ *g* = -0.45, a medium effect size).

Previous authors have claimed that DA strategies serve to reduce regret [1, 46, 78] and various studies of specific DA strategies have reported evidence in favour of this claim (status quo: [11, 15, 17]; action omission: [14, 50, 79]; inaction inertia: [40, 41]; choice delegation: [13, 80]; choice deferral: [81]). Our overall result is somewhat consistent with this claim: across all studies, DA is associated with lower levels of regret.

Regret in decision making often stems from the feeling of personal responsibility and self-blame for the outcome [1, 82]. As noted in the Introduction, DA strategies offer sources for blame other than decision-makers themselves, like existing norms (e.g., status quo), prior decisions (e.g., omission or inaction inertia), or other agents (e.g., delegation) [1]. Therefore, DA can reduce feelings of responsibility and self-blame and, thereby, regret [26, 50, 55, 79, 83]. However, only status quo preservation appears to reduce regret reliably, even though other DA strategies also offer other sources of blame. So it seems premature to state that DA *in general* reduces regret and that evasion of personal responsibility is sufficient to reduce regret. That said, there clearly is quite some variability in the direction and magnitude of the effect for this collection of strategies, and it is very likely that there will be conditions under which these strategies reduce regret.

We tested to what extent this variability could be accounted for by several pre-selected moderator variables. Only previous experience systematically moderated the association between decision avoidance and regret. As indicated by Zeelenberg and colleagues [15, 18], decision behaviours rarely occur in isolation, but rather in the context of previous decision outcomes. When the DA option previously led to a negative outcome, choosing the DA option again effectively means doing nothing to improve the situation. In addition, according to Kahneman and Miller’s Normal Theory [46], when the prior choice resulted in a negative experience, repeating the same choice is unreasonable and “abnormal” behaviour [15, 18]. In this case, the sense of personal responsibility for the negative outcome and counterfactual thinking may be amplified, because the decision maker feels that they should have known better. As a result, feelings of regret are enhanced. We note that the role of negative previous experience was only studied in the context of status quo preservation—this finding needs further exploration with other DA strategies.

### Status quo and regret reduction

There are several reasons why status quo maintenance may be particularly well-suited to protect against post-decision regret. First, regret stems from the comparison of the actual choice with a counterfactual (“what if…”) choice [46, 84]. The more extensively a decision maker engages in this comparison the more regret they are likely to experience. People who change the status quo are likely to have paid more attention to the available options and engaged in a more extensive comparison of the features of these options and their possible outcomes [17, 44, 85]. It is also possible that having more information about the options increases their comparability—the extent to which options may be compared along similar dimensions [86]. Van Dijk and Zeelenberg [87] claimed that regret is linked to the comparability between options. Choosing to maintain the status quo may be a way of limiting the comparison between the options and in this way reduce regret.

Second, there is evidence that regret is linked to the size of the choice set, with decision makers reporting more regret when the number of alternatives to choose from is larger [88, 89]. One reason for this link may be that with limited options, there is less of a sense of responsibility for the outcome. Those who change the status quo may have paid more attention to all the available options, thereby effectively increasing their choice set. Moreover, with a sense of increased responsibility may also come an element of fear of not being able to make the best choice given limited time and effort [89, 90]—after all, with more options to choose from there is more uncertainty about whether you have picked the best one. This fear may, in turn, lead to increased counterfactual thinking, which increases regret.

Third, Kahneman and Miller [46] suggested in their Norm Theory that emotional responses to the outcomes of decisions are amplified by “abnormal causes”. The extent to which a cause is (ab)normal may be evaluated in several ways [91] such as the similarity to previous behaviour [84], and the consistency with other people [22]. In this case, deviations from the status quo could be considered as psychologically abnormal causes which can amplify the feeling of regret.

### Open questions and future directions

One contribution of the current paper is that its findings raise several open questions to guide future research and/or theoretical development. First, there is insufficient evidence to estimate reliably the relationship between DA and regret for non-status quo strategies, because by far most of the studies have focused on status quo preservation. There was a reasonable number of effect sizes for action omission, but seven out of 13 of these came from one paper. There were no studies of choice deferral that met our selection criteria (or even the more relaxed criteria for qualitative synthesis), and the number of studies on inaction inertia and choice delegation was very small. Clearly then, we cannot get a precise pooled effect size for every DA strategy. We suggest future research is selectively directed to these under-represented DA strategies.

Second, our results suggest two types of publication bias. There was an asymmetry in favour of a more negative association in the funnel plot of the full sample (Fig 5). Moreover, in the full dataset about 30% of the effect sizes were outside the 99% confidence interval around the pooled effect size. This result may be indicative of a ‘file drawer problem’ [92] in that studies with small and/or non-significant effects are not written up, submitted or accepted for publication.

Third, on a more theoretical note, although status quo preservation and action omission are often aligned (i.e., inaction preserves the status quo; action alters the status quo), there are situations in which their relation is less clear-cut or in which they are misaligned (i.e., inaction changes the status quo; action preserves the status quo). In the case where action omission is not aligned with status quo preservation, it is not clear whether the decision maker engaged in DA, and the relationship between DA and regret is muddied. Suppose action omission changed the status quo and the decision maker experiences more regret [47]. If we think that the agent engaged in DA (action omission) we would conclude that DA *increases* regret (inconsistent with the overall hypothesis tested here). However, if we think that the agent chose to change the status quo, we would conclude that such a change increases regret (an effect that would be consistent with status quo preservation reducing regret, in line with the overall hypothesis tested here).

Some studies have begun to address how the relationship between DA and regret changes when status quo preservation and action omission are misaligned. For example, Feldman and Albarracín [22] created third-person scenarios in which either action or inaction is the norm (i.e., consistent with the status quo). When inaction is the norm, status quo and action omission are aligned. When action is the norm, status quo and action omission are misaligned. In the scenarios, participants were told about two agents; one of them chose action and the other one chose inaction. Participants were asked to indicate which agent experienced more regret. When action omission and status quo were aligned, participants attributed more regret to the agent who chose action and went against the norm. When status quo and action omission were misaligned so that action preserved the norm, this effect was weakened or even reversed. This example highlights the importance of considering the possible interaction between DA strategies.

Fourth, and finally, in previous experiments choosing a DA option has been regarded as DA behaviour, but of course it is entirely possible that the DA option is chosen after a period of effortful deliberation (or for a number of other reasons, such as the recognition heuristic). That is, DA behaviour is classified as such based on the choice *outcome*, rather than the choice *process*. Choosing the DA option may not always represent a strategy to minimise effort or defer responsibility. For example, when people choose the status quo option, they may have carefully compared all alternative options before sticking with the status quo. Therefore, future studies should consider not only the final choice but rather should focus on the decision making process when identifying whether and to what extent participants engage with DA.

In conclusion, this meta-analysis suggests that DA can reduce post-decision regret, although this overall effect appears small and may be subject to publication bias. However, the effect appears larger and robust for status quo preservation. The extent to which other DA strategies reduce regret and under what conditions is still an open question. Moreover, we need better ways of identifying the process by which an agent settles on a DA option, for example, using process tracing methodologies [93]. This analysis has identified a number of gaps in the literature and future research should aim at filling those gaps.

## Supporting information

S1 File Appendix. (DOCX)

S1 Table PRISMA 2020 checklist. (DOCX)
