# Supplementary material for: Decision avoidance and post-decision regret: A systematic review and meta-analysis
Source: PLoS One. 2023 Oct 13;18(10):e0292857. doi: 10.1371/journal.pone.0292857 (PMC10575496; doi:10.1371/journal.pone.0292857)

**Appendices**

**Text S1. Search strategies.**

In all databases but JSTOR, the search string is: (“decision avoidance” OR “status quo” OR “default bias” OR “omission” OR “option fixation” OR “inaction inertia” OR “deferr*” OR “delegat*”) AND (“counterfactual thinking” OR “regret”). In JSTOR, the search string is: (ab:(decision avoidance or status quo or default bias or omission or option fixation or inaction inertia or deferr* or delegat*) AND ab:(Counterfactual Thinking OR regret)) AND la:(eng OR en).

**Table S1**

*Narrative review for articles not eligible for meta-analysis*

| **Decision Avoidance** |  | **Author** | **Paradigm** | **Example cover story** | **Results** | **Direction** |
| --- | --- | --- | --- | --- | --- | --- |
| Status Quo |  | Tsiros & Mittal (2000) | scenario | Buy laptop, switching brand or changed brand | More regret for action (i.e., switching brands) than keeping the status quo (i.e., maintaining the same brand) | DA reduces regret |
|  |  | Mannetti et al (2007) | scenario | Buy laptop, switching brand or changed brand | Less regret for maintaining status quo compared to deciding to change, but only for participants with high need for closure | DA reduces regret (partial support) |
|  |  | Karadogan (2010) | scenario | Football coach change/don’t change the team | More regret for changing status-quo than maintaining | DA reduces regret |
|  |  | Nicolle et al (2011) | status-quo rejection task | Participants judging whether a target ball, landing on a simulated tennis court, was “IN” (overlapping the line) or “OUT”. Participants started each trial by holding the key corresponding to their choice on the previous trial (or holding “IN” on the very first trial). | Regret was higher when errors arise from rejection rather than acceptance of a status quo option (their previous choice) | DA reduces regret |
|  |  | El Zein & Bahrami (2020) | computer lottery task | Lottery Game: Participants can choose whether to play this game on their own or with four other people. When choosing to play with four other people, the lottery choice was based on the choices of the majority and not necessarily the subjects' own choices. | A group decision (i.e., playing a lottery game with four other people) produced less regret than one's own decision. | DA reduces regret |
|  |  | Avni-Babad (2003) | scenario | Stock market game: choose between keeping shares in a company or switching to a different company. | Greater regret for action than inaction. | DA reduces regret |
| Omission |  | Hattiangadi et al (1995) | Questionnaire at end of lives | Questionnaire at end of lives asking what had regretted. Some answers reflected omission and some commission | Many more inaction regrets reported | DA increases regret |
|  |  | Gilovich & Medvec (1994) | Questionnaire for daily decision | Questions about which actions or inactions resulted in the greatest regret. | Twenty-one of the 30 respondents (70%) expressed greater regret over their biggest failure to act | DA increases regret |
|  |  | Deuskar et al (2020) | existing dataset | Dataset from Chinese stock markets | Regret was stronger following action rather than inaction and an unusual strategy for the investor | DA increases regret |
| Delegation |  | Ordonez & Connolly (2000) | scenario | Undergraduates re-assigned to different course by themselves or by computer | More regret attributed to self-choosers over computer assigned (choice delegation) | DA reduces regret |

**Table S2a**

*Overview of 13 Articles (59 effect sizes) on Decision Avoidance and Regret.*

| **Decision avoidance** | **Author (year)** | **Study (condition)** | **Region** | | **Age** | **Gender (% female)** | **Timing of regret measurement** | **Outcome** | **Previous experience** | | **Within-subject** |
| --- | --- | --- | --- | --- | --- | --- | --- | --- | --- | --- | --- |
| Status quo | Kirkebøen et al. (2013) | Study 1 (pre-outcome regret) | | Europe | 25.9 | 0.73 | pre-outcome | unknown | | control | between |
|  |  | Study 1 (post-outcome regret) | | Europe | 25.9 | 0.73 | post-outcome | mixed | | control | between |
|  |  | Study 1 (long-term regret) | | Europe | 25.9 | 0.73 | long-term | mixed | | control | between |
|  |  | Study 2 (pre-outcome regret) | | Europe | 25.9 | 0.73 | pre-outcome | unknown | | control | between |
|  |  | Study 2 (post-outcome regret) | | Europe | 25.6 | 0.27 | post-outcome | mixed | | control | between |
|  |  | Study 2 (long-term regret) | | Europe | 25.9 | 0.73 | long-term | mixed | | control | between |
|  |  | Study 3 | | Europe | 25.7 | 0.81 | post-outcome | negative | | control | between |
|  | Zeelenberg et al. (2002) | Study 2 (positive previous experience) | | Europe | NA | 0.59 | post-outcome | negative | | positive | within |
|  |  | Study 2 (absent previous experience) | | Europe | NA | 0.58 | post-outcome | negative | | control | within |
|  |  | Study 2 (negative previous experience) | | Europe | NA | 0.57 | post-outcome | negative | | negative | within |
|  | Itzkin et al.  (2016) | Scenario 3 | | Asia | 39 | 0.52 | post-outcome | negative | | control | within |
|  |  | Scenario 4 | | Asia | 39 | 0.52 | post-outcome | negative | | control | within |
|  |  | Scenario 5 | | Asia | 39 | 0.52 | post-outcome | negative | | control | within |
|  |  | Scenario 6 | | Asia | 39 | 0.52 | post-outcome | negative | | control | within |
|  | Feeney & Handley (2006) | Study 1 (control) | | Europe | NA | NA | post-outcome | mixed | | control | within |
|  |  | Study 1 (actor 1st) | | Europe | NA | NA | post-outcome | mixed | | control | within |
|  |  | Study 1 (actor 2nd) | | Europe | NA | NA | post-outcome | mixed | | control | within |
|  |  | Study 2 (control) | | Europe | NA | NA | post-outcome | mixed | | control | within |
|  |  | Study 2 (actor 1st) | | Europe | NA | NA | post-outcome | mixed | | control | within |
|  |  | Study 2 (actor 2nd) | | Europe | NA | NA | post-outcome | mixed | | control | within |
|  |  | Study 3 (similar) | | Europe | NA | NA | post-outcome | mixed | | control | within |
|  |  | Study 3 (dissimilar) | | Europe | NA | NA | post-outcome | mixed | | control | within |
|  | Inman & Zeelenberg (2002) | Study 1 (control previous experience) | | Asia | NA | NA | post-outcome | negative | | control | between |
|  |  | Study 1 (positive previous experience) | | Asia | NA | NA | post-outcome | negative | | positive | between |
|  |  | Study 2 (negative previous experience) | | Asia | NA | NA | post-outcome | negative | | negative | between |
|  |  | Study 2 (high history: control previous experience) | | Asia | NA | NA | post-outcome | negative | | control | between |
|  |  | Study 2 (high history: positive previous experience) | | Asia | NA | NA | post-outcome | negative | | positive | between |
|  |  | Study 2 (high history: negative previous experience) | | Asia | NA | NA | post-outcome | negative | | negative | between |
|  |  | Study 2 (low history: control previous experience) | | Asia | NA | NA | post-outcome | negative | | control | between |
|  |  | Study 2 (low history: positive previous experience) | | Asia | NA | NA | post-outcome | negative | | positive | between |
|  |  | Study 2 (low history: negative previous experience) | | Asia | NA | NA | post-outcome | negative | | negative | between |
|  |  | Study 4 (strong) | | Asia | NA | NA | post-outcome | negative | | control | between |
|  |  | Study 4 (weak) | | Asia | NA | NA | post-outcome | negative | | control | between |
| Omission | Sevdalis et al.  (2006) | Study 2 (basic purchasing) | | Europe | 20.9 | 0.61 | NA | not exist | | control | within |
|  | Abendroth & Diehl  (2006) | Study 2 (short-term regret) | | America | NA | NA | pre-outcome | unknown | | control | between |
|  |  | Study 2 (long-term regret high utility) | | America | NA | NA | post-outcome | positive | | control | between |
|  |  | Study 2 (long-term regret low utility) | | America | NA | NA | post-outcome | negative | | control | between |
|  |  | Study 3 (short-term regret limited) | | America | NA | NA | pre-outcome | unknown | | control | between |
|  |  | Study 3 (long-term regret limited) | | America | NA | NA | post-outcome | negative | | control | between |
|  |  | Study 3 (short-term regret unlimited) | | America | NA | NA | pre-outcome | unknown | | control | between |
|  |  | Study 3 (long-term regret unlimited) | | America | NA | NA | post-outcome | negative | | control | between |
|  | Seiler et al.  (2008) | Only one study | | NA | 27.5 | 0.41 | post-outcome | neutral | | control | within |
|  | Seta et al. (2001) | Study 1 risker seeker | | America | NA | 0.91 | post-outcome | negative | | control | between |
|  |  | Study 1 risker avoider | | America | NA | 0.91 | post-outcome | negative | | control | between |
|  |  | Study 3 active mood | | America | NA | 1.00 | post-outcome | negative | | control | between |
|  |  | Study 3 inactive mood | | America | NA | 1.00 | post-outcome | negative | | control | between |
|  |  | Study 4 active mood | | America | NA | 0.90 | post-outcome | neutral | | control | between |
|  |  | Study 4 inavtive mood | | America | NA | 0.90 | post-outcome | neutral | | control | between |
|  | Jamison et al. (2020) | Scenario 1 harm outcome | | America | 36.4 | 0.50 | post-outcome | negative | | control | between |
|  |  | Scenario 1 no-harm outcome | | America | 36.4 | 0.50 | post-outcome | neutral | | control | between |
|  |  | Scenario 2 harm outcome | | America | 36.4 | 0.5 | post-outcome | negative | | control | between |
|  |  | Scenario 2 no-harm outcome | | America | 36.4 | 0.5 | post-outcome | neutral | | control | between |
|  | Feldman (2020) | Experiment 2 (control norms) | | America | 36.6 | 0.58 | post-outcome | negative | | control | between |
| Inaction Inertia | Sevdalis et al.  (2006) | Study 1 | | Europe | 21.0 | 0.67 | post-outcome | not exist | | control | within |
|  |  | Study 2 (large difference) | | Europe | 20.9 | 0.61 | post-outcome | not exist | | control | within |
|  |  | Study 2 (small difference) | | Europe | 20.9 | 0.61 | post-outcome | not exist | | control | within |
|  | Lee (2015) | Only one study | | America | 21.5 | 0.56 | post-outcome | not exist | | control | within |
| Delegation | Wagner et al.  (2012) | Decision for self | | Europe | 23.6 | 0.45 | post-outcome | negative | | control | within |
|  |  | Decision for other | | Europe | 23.6 | 0.45 | post-outcome | negative | | control | within |

**Table S2b**

*Further Information of 13 Included Articles on Decision Avoidance and Regret.*

| **Author** | **Year** | **Decision avoidance strategy** | **Paradigm** | **Cover story** | **Regret measurement** |
| --- | --- | --- | --- | --- | --- |
| Kirkebøen et al. | 2013 | Status Quo | ultimatum | In the ultimatum game, participants make proposals that can be accepted or rejected by another player, and the player's decision may lead to acceptance or regret. Participants have the flexibility to either modify their initial proposal or retain it. | 2 items; 7-point |
| Zeelenberg et al. | 2002 | Status Quo | scenario | In the context of football, coaches face the decision of whether to change or maintain their players' lineup based on the team's performance, such as winning, losing, or tying a match. | 1 item; 7-point |
| Itzkin et al. | 2016 | Status Quo | scenario | In six different scenarios, individuals have the option to either maintain the status quo (inaction) or initiate a change (action). | 2 items; 101-point |
| Feeney & Handley | 2006 | Status Quo | scenario | Paticipants face the decision of either transferring to another university or remaining at their current one. | 1 item; 101-point |
| Inman & Zeelenberg | 2002 | Status Quo | scenario | The decision involves either switching to a different airline, backpack, or hotel, or sticking with the current choice. | 3 items; 10-point |
| Feldman | 2020 | Status Quo | scenario | The experiment manipulated social norms and participants had three options: taking action, doing nothing, or choosing neither of these options. Following this, the scenario involved a football coach who had to decide whether to change or not change a player based on the outcome of the game (win, loss, or tie). | 1 item; 6-point |
| Sevdalis et al. | 2006 | Omission/Inaction Inertia | scenario | The scenario of inaction inertia refers to the tendency to maintain the current state or refrain from taking action. | 1 item;10-point |
| Abendroth & Diehl | 2006 | Omission | scenario | The scenario involves attending a concert by their favorite artist and deciding whether to purchase the official shirt or not. | 1 item; 7-point |
| Seiler et al. | 2008 | Omission | scenario | The scenario pertains to an investment in a property, and the decision revolves around either selling it earlier or keeping it for a longer duration. | 1 item; 9-point |
| Seta et al. | 2001 | Omission | scenario | The scenario involves the decision to either retain the original company shares or switch to shares of another company. | 1 item; 101-point |
| Jamison et al. | 2020 | Omission | scenario | Ellen's behavior had two options: either she acted to harm Peter directly (commission) or she refrained from taking action that could have prevented harm to Peter (omission). The outcome of this situation was that Peter either suffered harm by being charged for the accident or was not harmed as he was not charged. | 1 item; 7-point |
| Lee | 2015 | Inaction Inertia | scenario | The scenario of inaction inertia refers to the tendency to maintain the current state or refrain from taking action. | 1 item; 7-point |
| Wagner et al. | 2012 | Delegation | computer gambling task | The study examined participants' decision-making in a lottery gamble. Participants were presented with a choice between taking the gamble or not taking it. | 1 item; 11-point |

**Text S2. Effect sizes.**

To address the issue that we treated all the effect sizes as if they came from a between-subjects design, we implemented a (crude) correction of the pooled standard deviation (see the formula of Hedges’ *g_av_*; Hedges & Olkin, 1985; Lakens, 2013) and compared the estimates of Hedges’ *g* (equation 1; Hedges & Olkin, 1985) with this Hedges’ *g_av_* (equation 2; Hedges & Olkin, 1985) for the studies of within-subject design.

The Hedges’ *g* formula is (between-subject design, used in this meta-analysis):

$\mathrm{Hedge}s^{'} g= \frac{M_{1}-M_{2}}{{SD}_{pooled}} \times(1 - \frac{3}{4\left( n_{1} + n_{2} \right)-9})$ (1)

$${SD}_{pooled}= \sqrt{\frac{\left( n_{1}-1 \right){SD}_{1}^{2}+ \left( n_{2}-1 \right){SD}_{2}^{2}}{n_{1}+ n_{2}-2}}$$

The Hedges’ *g_av_* formula is (within-subject design):

$Hedges^{'} g_{av}= \frac{M_{1} - M_{2}}{\frac{\left( {SD}_{1} + {SD}_{2} \right)}{2}}\times\left( 1 - \frac{3}{8n-9} \right)$(2)

Figure S1 shows the sub-set of within-subject effect sizes, with the effect size calculated as either the standard Hedges’ *g* or Hedges’ *g_av_*. It is clear that the differences are negligible, which is not surprising given that when the two “group” sizes are equal (which is necessarily the case in a within-subjects design), the pooled SD and the average SD are very similar. Nevertheless, where there are small differences, the “between-subjects” approximation to the within-subject effect size is smaller—making this approximation a relatively conservative measure.

**Figure S1**

*A comparison of* Hedges’ *g and* Hedges’ *g_av_ for the effects that were measured within-subjects.*


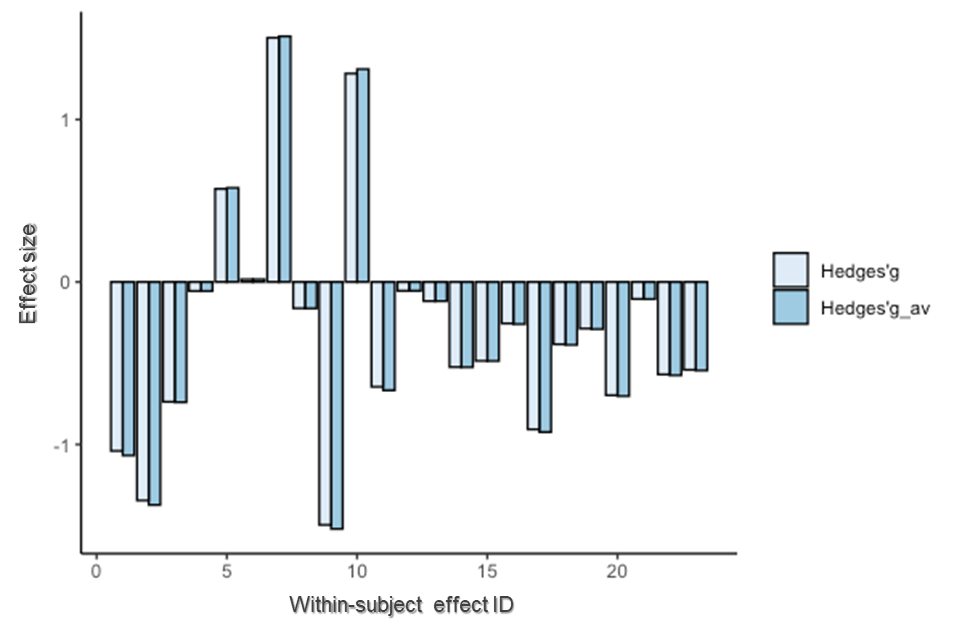

Supplement: S1 File — (DOCX) [file pone.0292857.s001.docx]
